# A Common Genetic Variant at 15q25 Modifies the Associations of Maternal Smoking during Pregnancy with Fetal Growth: The Generation R Study

**DOI:** 10.1371/journal.pone.0034584

**Published:** 2012-04-04

**Authors:** Elisabeth T. M. Leermakers, H. Rob Taal, Rachel Bakker, Eric A. P. Steegers, Albert Hofman, Vincent W. V. Jaddoe

**Affiliations:** 1 The Generation R Study Group, Erasmus Medical Center, Rotterdam, the Netherlands; 2 Department of Epidemiology, Erasmus Medical Center, Rotterdam, the Netherlands; 3 Department of Paediatrics, Erasmus Medical Center, Rotterdam, the Netherlands; 4 Department of Obstetrics and Gynaecology, Erasmus Medical Center, Rotterdam, the Netherlands; University of Southampton, United Kingdom

## Abstract

**Objective:**

Maternal smoking during pregnancy is associated with fetal growth retardation. We examined whether a common genetic variant at chromosome 15q25 (rs1051730), which is known to be involved in nicotine metabolism, modifies the associations of maternal smoking with fetal growth characteristics.

**Methods:**

This study was performed in 3,563 European mothers participating in a population-based prospective cohort study from early pregnancy onwards. Smoking was assessed by postal questionnaires and fetal growth characteristics were measured by ultrasound examinations in each trimester of pregnancy.

**Results:**

Among mothers who did not smoke during pregnancy (82.9%), maternal rs1051730 was not consistently associated with any fetal growth characteristic. Among mothers who continued smoking during pregnancy (17.1%), maternal rs1051730 was not associated with head circumference. The T-allele of maternal rs1051730 was associated with a smaller second and third trimester fetal femur length [differences −0.23 mm (95%CI −0.45 to −0.00) and −0.41 mm (95%CI −0.69 to −0.13), respectively] and a smaller birth length [difference −2.61 mm (95%CI −5.32 to 0.11)]. The maternal T-allele of rs1051730 was associated with a lower third trimester estimated fetal weight [difference −33 grams (95%CI −55 to −10)], and tended to be associated with birth weight [difference −38 grams (95%CI −89 to 13)]. This association persisted after adjustment for smoking quantity.

**Conclusions:**

Our results suggest that maternal rs1051730 genotype modifies the associations of maternal smoking during pregnancy with impaired fetal growth in length and weight. These results should be considered as hypothesis generating and indicate the need for large-scale genome wide association studies focusing on gene – fetal smoke exposure interactions.

## Introduction

Maternal smoking during pregnancy is strongly associated with increased risks of preterm birth or a small size for gestational age at birth [1]. As compared to children of mothers who did not smoke during pregnancy, those of mothers who smoked during pregnancy have a 100 to 200 grams lower birth weight [1]. It has been estimated that, in Western countries, 30 percent of children with low birth weight can be explained by exposure to tobacco smoke during pregnancy [2]–[3]. The effects of maternal smoking on fetal growth differ between individuals. These differences in effects might be explained by maternal genetic predisposition. A recent genome wide association study meta-analysis identified a common genetic variant, rs1051730, located within the 15q25 nicotinic acetylcholine receptor gene cluster (*CHRNA5-CHRNA3-CHRNB4*), to be associated with smoking quantity [4]. Smokers with a risk allele (T-allele) of rs1051730 seem to have higher blood levels of nicotine, compared to smokers without the T-allele [5]. Another study showed that among mothers who smoked during pregnancy, each additional copy of the maternal T-allele of rs1051730 resulted in a 28 grams lower birth weight in the offspring [6], though this effect was not significant. These findings suggest that rs1051730 might be a common genetic variant leading to an increased susceptibility of the adverse effects of maternal smoking on fetal growth and development.

Therefore, we explored in a population-based prospective cohort study among 3,563 European mothers, whether maternal rs1051730 modifies the associations of maternal smoking during pregnancy with fetal growth characteristics in different trimesters.

## Results

In our population, 610 (17.1%) mothers continued smoking during pregnancy (Table 1). Mean maternal age was 31.1 years. As compared to mothers who did not smoke during pregnancy, mothers who smoked during pregnancy were shorter, had higher weight and were lower educated. Children of mothers who smoked during pregnancy had a lower birth weight than children of mothers who did not smoke. Distribution of maternal genotype of rs1051730 was different between non-smokers and continued smokers. Genotype was not associated with smoking quantity. Subject characteristics per maternal genotype group among continued smokers are given in Table S1.

**Table 1:** Subject characteristics of the mothers per smoking status (n = 3,563)^1^.

	Total	Non-smokers	Continued smokers
	N = 3,563	N = 2,953 (82.9%)	N = 610 (17.1%)
**Mother**			
Age (years)	31.1 (4.5)	31.4 (4.2)	29.7 (5.6)**
Gestational age at enrollment^2^ (weeks)	13.6 (10.1 to 23.2)	13.6 (10.2 to 22.9)	14.1 (9.5 to 29.4)**
Height (cm)	170.3 (6.5)	170.4 (6.5)	169.3 (6.5)**
Weight (kg)	70.2 (12.6)	70.0 (12.2)	71.2 (14.0)*
Body mass index (kg/m^2^)	24.2 (4.1)	24.1 (4.0)	24.8 (4.5)**
Parity (% nullipara)	59.8	60.4	57.4
Highest education finished (%)			
Primary school	4.4	2.6	13.3**
Secondary school	38.5	33.7	62.5**
Higher education	57.1	63.8	24.2**
Alcohol consumption during pregnancy (% yes)	65.3	66.2	61.3*
Number of cigarettes smoked (%)			
<5 per day	NA	NA	45.2
5–10 per day	NA	NA	32.7
>10 per day	NA	NA	22.0
Genotype (%)			
G/G	45.4	46.3	41.0*
G/T	43.1	42.2	47.4*
T/T	11.6	11.5	11.6
**Child**			
Gestational age at birth^2^ (weeks)	40.1 (35.6 to 42.3)	40.3 (35.8 to 42.3)	40.0 (34.8 to 42.3)**
Birth weight (grams)	3476 (558)	3514 (553)	3290 (546)**
Sex (% Boys)	49.6	49.1	52.5

1 Values are means (SD) or percentages.

2 Median (95% range).

Differences in distributions between groups were evaluated using a Student T-test for continuous variables and Chi-square tests for categorical variables.

* *P*-value<0.05;

** *P*-value<0.01.

In the total population, we did not observe associations of the maternal T-allele of rs1051730 with fetal growth characteristics (Table 2). Among mothers who continued smoking, we observed non-significant tendencies toward smaller (fetal) head circumference for each additional copy of the maternal T-allele of rs1051730. The T-allele of maternal rs1051730 was associated with a smaller second and third trimester fetal femur length [differences −0.23 mm (95%CI −0.45 to −0.00) and −0.41 mm (95%CI −0.69 to −0.13), respectively] and tended to be associated with a smaller birth length [difference −2.61 mm (95%CI −5.32 to 0.11)] (Table 2). For all fetal length measures, we found a significant interaction between maternal smoking status and maternal genotype of rs1051730 [*P_interaction_*<0.05]. Among mothers who smoked during pregnancy, the T-allele was associated with a lower third trimester estimated fetal weight [difference −32.7 grams (95%CI −55.4 to −10.0)], and tended to be associated with lower birth weight [difference −38.1 grams (95%CI −89.2 to 12.9)]. Among the mothers who smoked during pregnancy, those who had a T/T genotype gave birth to children with a birth weight that was 117 grams lower (95% CI −229 to −4 grams), as compared to children whose mothers had a G/G genotype. We found a significant interaction between maternal smoking status and maternal genotype of rs1051730 for fetal weight at all time points [*P_interaction_*<0.05].

**Table 2:** Cross-sectional associations of maternal rs1051730 genotype with fetal growth characteristics in different trimesters^1^ (n = 3,563).

	Second trimester	Third trimester	Birth
	Head circumference	Head circumference	Head circumference
	Difference (95% CI) (mm)	Difference (95% CI) (mm)	Difference (95% CI) (mm)
**Total Group** *N = 3,546*	−0.26 (−0.56 to 0.04)	0.11 (−0.33 to 0.55)	0.16 (−0.63 to 0.95)
Non-smokers *N = 2,940*	−0.23 (−0.55 to 0.10)	0.23 (−0.25 to 0.71)	0.31 (−0.55 to 1.17)
Smokers *N = 606*	−0.43 (−1.21 to 0.35)	−0.44 (−1.55 to 0.66)	−0.67 (−2.71 to 1.37)
**Interaction** 2	*P = 0.62*	*P = 0.24*	*P = 0.39*

	Femur length	Femur length	Body length
	Difference (95% CI) (mm)	Difference (95% CI) (mm)	Difference (95% CI) (mm)
**Total Group** *N = 3,549*	0.00 (−0.09 to 0.09)	−0.01 (−0.12 to 0.11)	0.33 (−0.77 to 1.42)
Non-smokers *N = 2,943*	0.04 (−0.05 to 0.14)	0.08 (−0.04 to 0.20)	0.82 (−0.38 to 2.02)
Smokers *N = 606*	−0.23 (−0.45 to −0.00)*	−0.41 (−0.69 to −0.13)**	−2.61 (−5.32 to 0.11)
**Interaction** 2	*P = 0.03*	*P<0.01*	*P = 0.04*

	Estimated fetal weight	Estimated fetal weight	Weight
	Difference (95% CI) (g)	Difference (95% CI) (g)	Difference (95% CI) (g)
**Total Group** *N = 3,563*	0.28 (−1.88 to 2.43)	3.05 (−6.23 to 12.32)	15.95 (−5.59 to 37.48)
Non-smokers *N = 2,953*	1.36 (−0.99 to 3.71)	10.37 (0.22 to 20.51)*	26.81 (3.05 to 50.57)*
Smokers *N = 610*	−5.36 (−10.73 to 0.01)	−32.66 (−55.36 to −9.97)**	−38.13 (−89.16 to 12.90)
**Interaction** 2	*P = 0.02*	*P<0.01*	*P = 0.03*

1 Effect estimates (with 95% confidence interval) reflect the differences in each growth characteristic for each additional copy of the T-allele of rs1051730 (assuming an additive model).

2 Interaction term = maternal genotype×smoking status.

* *P*-value<0.05;

** *P*-value<0.01.

All analyses were adjusted for gestational age at visit and sex. Analyses in the total group were additionally adjusted for smoking status (yes, no). Birth length and head circumference at birth were additionally adjusted for source of the birth measurements.

**Figure 1:**
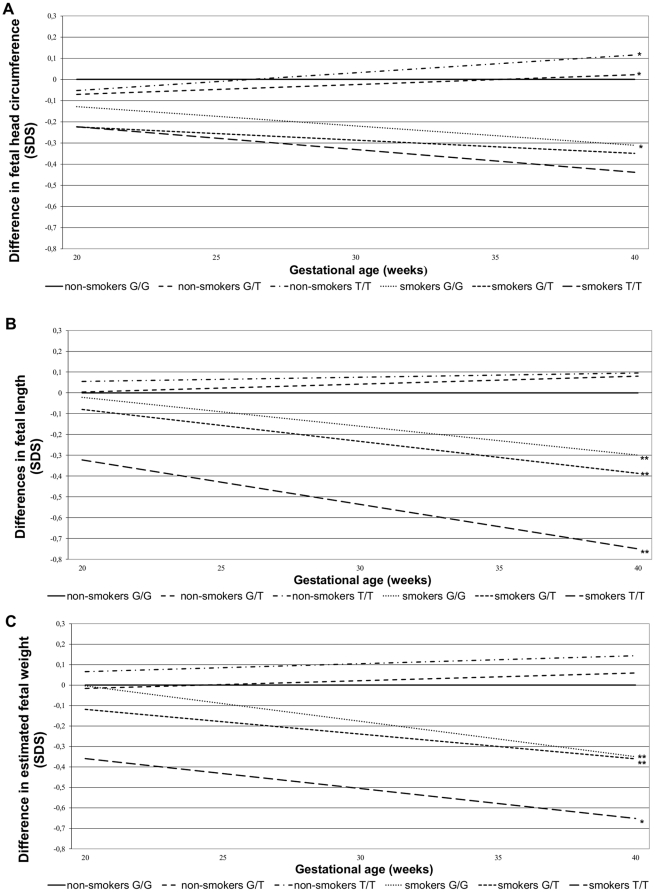
Effect of maternal genotype and smoking status on fetal growth characteristics (SD scores)^1^. ^1^Values are based on repeated linear regression models and reflect the differences in growth in gender and gestational age specific standard deviation scores (SDS) between the number of risk alleles and smoking status compared to the reference group (genotype G/G, non-smokers). Estimates are given in the Supplementary Table S2. *P-value<0.05; **P-value<0.01. (Figure 1a = head circumference growth, Figure 1b = length growth, Figure 1c = weight growth).

In Figure 1, the results of maternal genotype of rs1051730 per smoking status with longitudinal fetal growth patterns are presented as difference in standard deviation scores, adjusted for gestational age and sex. This gives the relative effect in different periods of pregnancy. All genotype categories of mothers who smoked during pregnancy had smaller fetal head circumference, length and weight measures, as compared to the mothers who did not smoke during pregnancy. Among mothers who did not smoke during pregnancy, the maternal T-allele of rs1051730 had a positive effect on head circumference growth, but was not significantly associated with length and weight growth. Among mothers who smoked during pregnancy, the T-allele was associated with the smallest head circumference, length and weight growth. The effects seem to be larger for length and weight than for head circumference. The specific coefficients of the gestational age independent and dependent differences (interaction smoke status, rs1051730 genotype and gestational age) for these models are given in Table S2.

We performed several sensitivity analyses. First, results of the analyses with a dominant genetic model are shown in Table S3. Second, additional analyses showed that the fetal genotype of rs1051730, was not associated with any fetal growth characteristic after adjustment for the maternal genotype (Table S4). Furthermore, adjustment for maternal smoking quantity did not affect our results (Table S5). Additional adjustment for maternal age, body mass index at enrollment, parity, educational level and alcohol consumption slightly attenuated the effect of maternal rs1051730, although the directions of the effects remained similar (Table S6). In these fully adjusted models, the interaction of maternal smoking and genotype remained significant in third trimester of pregnancy.

## Discussion

### Main findings

Results from this prospective population-based cohort study suggest that maternal genotype of the common genetic variant, rs1051730, located within the 15q25 nicotinic acetylcholine receptor gene cluster (*CHRNA5-CHRNA3-CHRNB4*), influences the susceptibility of impaired fetal growth by maternal smoking. Among mothers who smoked during pregnancy, the T-allele resulted in smaller femur length and lower estimated fetal weight from second trimester onwards.

### Methodological considerations

An important strength of this study was the prospective design and the large sample size of 3,563 participants being studied from early pregnancy onwards. To our knowledge, this study is the first study that examined the associations of maternal smoking during pregnancy, and rs1051730, with fetal growth characteristics in different trimesters of pregnancy.

A potential limitation of our study is that information about smoking during pregnancy was collected by questionnaires. Although assessing smoking during pregnancy by questionnaire seems to be a valid method, misclassification may occur [7]. To overcome these limitations, previous studies have used biomarkers of tobacco exposure, including cotinine -a metabolite of nicotine- in maternal urine samples [8]–[9]. However, it has been demonstrated that use of cotinine levels is not superior to the use of self-reporting questionnaires in studying the effect of maternal smoking in pregnancy on birth weight [10]. In general, confounding is not likely to be a major issue in gene - outcome association studies. The unadjusted and adjusted models focused on the associations of rs1051730 genotype with fetal growth characteristics did not show large differences in effect estimates. However, smoking quantity might be a relevant confounder in our study, since rs1051730 is known to be associated with smoking quantity [4]. We adjusted the analyses for the number of cigarettes smoked per day reported in first trimester, which did not change the effect estimates. Furthermore, in our population of mothers who smoked during pregnancy, smoking quantity was not associated with rs1051730 genotype in neither first, second nor third trimester. We also performed a sensitivity analysis using second and third trimester smoking quantity instead of first trimester smoking quantity in the models, which did not alter our results. Therefore it seems unlikely that smoking quantity has biased our results.

Another potential limitation could be that women who quitted smoking when pregnancy was known (first trimester only) were considered as non-smokers in our analyses. This could have resulted in biased effect estimates if fetal growth would be different between quitted smokers and non-smokers. However, we have previously reported that first trimester smoking only does not affect second and third trimester fetal growth [11]. We performed a sensitivity analysis in which we excluded women who quitted smoking in first trimester, which did not change our results (data not shown).

We established gestational age by ultrasound. By using this method, growth variation of the fetal characteristics used for pregnancy dating is assumed to be zero. In our study, first trimester crown-rump length and biparietal diameter were used for pregnancy dating but not for assessing fetal growth. Since pregnancy dating characteristics and growth characteristics are correlated throughout pregnancy, growth variation in head circumference, abdominal circumference, and femur length may be reduced by dating the pregnancy on the other fetal characteristics. This may have led to underestimation of our effect estimates. However, we expect this effect to be small in our results. This underestimation will consequently be lowest in women included in the first trimester (54.7% of the population for analysis). We performed a sensitivity analysis using only these women, but results did not change (data not shown).

### Maternal rs1051730 genotype, smoking during pregnancy and fetal growth

The effects of maternal smoking status during pregnancy on fetal growth have been studied previously, showing that among mothers who continued smoking during pregnancy, children had smaller femur length from second trimester onwards and smaller head circumferences from third trimester onwards, as compared to children from mothers who did not smoke during pregnancy [11]. As compared to children of mothers who did not smoke during pregnancy, those of mothers who smoked during pregnancy have a 100 to 200 grams lower birth weight [1]. This fetal growth restriction might be caused by developmental adaptations in placental vasculature and adaptations in fetal arterial resistance. A study that investigated these adaptations showed an increased umbilical resistance artery pulsatility index (PI) in pregnant women who smoked, which indicates higher feto-placental resistance. Also, a higher umbilical artery PI was associated with lower estimated fetal weight and birth weight [12].

The effects of maternal smoking on fetal growth are known to differ between individuals. We have previously demonstrated that maternal first trimester folic acid supplementation use reduces the adverse effects of maternal smoking during pregnancy on fetal growth [13]. Differences in effects of maternal smoking on fetal growth might also be explained by maternal genetic variation. We hypothesized that maternal genotype of rs1051730 modifies the association of maternal smoking during pregnancy with fetal growth. The T-allele of rs1051730 is associated with higher smoking quantity and higher nicotine levels in adults [4]–[5], [14]. A previous study showed that children from mothers who smoked during pregnancy, had a tendency towards a 28 grams lower birth weight per T-allele. No association was found among mothers who did not smoke during pregnancy [6]. In line with this previous study, we observed that among mothers who smoked during pregnancy, the T-allele resulted in smaller femur length and lower estimated fetal weight from second trimester onwards. We did not observe any effect on fetal head circumference. The effects of maternal smoking in fetal head circumference seem not to be affected by rs1051730. Our results also suggest that rs1051730 does not have a direct effect on birth weight, but needs environmental factors to exert its effect. Surprisingly, among non-smokers, we found a positive effect on fetal weight with each copy of the risk allele. We can not clearly explain this finding. It might me hypothesized that specific gene-environmental interactions, such as DNA methylation, changes the effects of specific genetic variants [15]. In a recent meta-analysis, the risk allele of rs1051730 was associated with a lower body mass index among smokers, while some studies of this meta-analysis showed a higher body mass index among non-smokers with the risk allele. However, the overall effect of the meta-analysis showed no effect of genotype among non-smokers [16]. We think that further research is necessary to explore the different effects of risk alleles of rs1051730 on various health outcomes among both smokers and non-smokers.

The mechanism through which rs1051730 modifies the associations between maternal smoking and fetal growth is yet unknown. Rs1051730 is located in the nicotinic acetylcholine receptor gene cluster (*CHRNA5-CHRNA3-CHRNB4*) on chromosome 15q25. This locus has been investigated previously and common genetic variants in high linkage disequilibrium with rs1051730 (HapMap r^2^>0.8) were associated with higher circulating cotinine levels, but not with number of cigarettes smoked per day [14]. A previous study that assessed the association of rs1051730 with the risk of lung cancer, observed that subjects with the T-allele had higher mean nicotine equivalents. In this study, the T-allele seemed to behave as a dominant model after adjustment for smoking quantity [5]. The authors therefore concluded that T-allele carriers extract a greater amount of nicotine per cigarette and are exposed to a higher internal dose of tobacco-specific nitrosamine, as compared to subjects without the T-allele [5]. Whether this is caused by more intense smoking or by a change in the nicotine metabolism, is not clear yet. Other studies suggested an additive genetic model for the T-allele of rs1051730 [6], [14], which was also most compliant with our data. Thus our results suggest that children of mothers who smoked during pregnancy are exposed to higher levels of nicotine when the mother has a T-allele of rs1051730. Therefore, these children might be more susceptible to the adverse effects of maternal smoking during pregnancy. Further research is necessary to determine the exact mechanism and underlying genetic model.

Gene-environment interaction studies are of increasing importance in modern medicine. Although smoking cessation should be advised to all pregnant women, a genetic risk profile might help us to target more effectively those at higher risk of growth retardation and adverse pregnancy outcomes. Further research is needed to identify groups at risk and develop new preventive strategies.

### Conclusions

Our results suggest that maternal genotype of the 15q25 variant rs1051730 influences the susceptibility of impaired fetal growth in length and weight by maternal smoking. This association of rs1051730 with fetal growth was present in mothers who continued smoking during pregnancy, but not in mothers who did not smoke during pregnancy. These results should be considered as hypothesis generating and indicate the need for large-scale genome wide association studies focusing on gene – fetal smoke exposure interactions.

## Materials and Methods

### Design

This study was embedded in the Generation R Study, a population-based prospective cohort study from early fetal life onwards in Rotterdam, the Netherlands, which has been described previously [17]–[18]. Enrollment was aimed at early pregnancy but was allowed until delivery. Extensive assessments focused on fetal growth and its main determinants were performed in each trimester of pregnancy. All children were born between April 2002 and January 2006. The study has been approved by the Medical Ethical Committee of the Erasmus Medical Center in Rotterdam (MEC 198.782/2001/31). Written informed consent was obtained from all participants.

### Population for analysis

**Figure 2:**
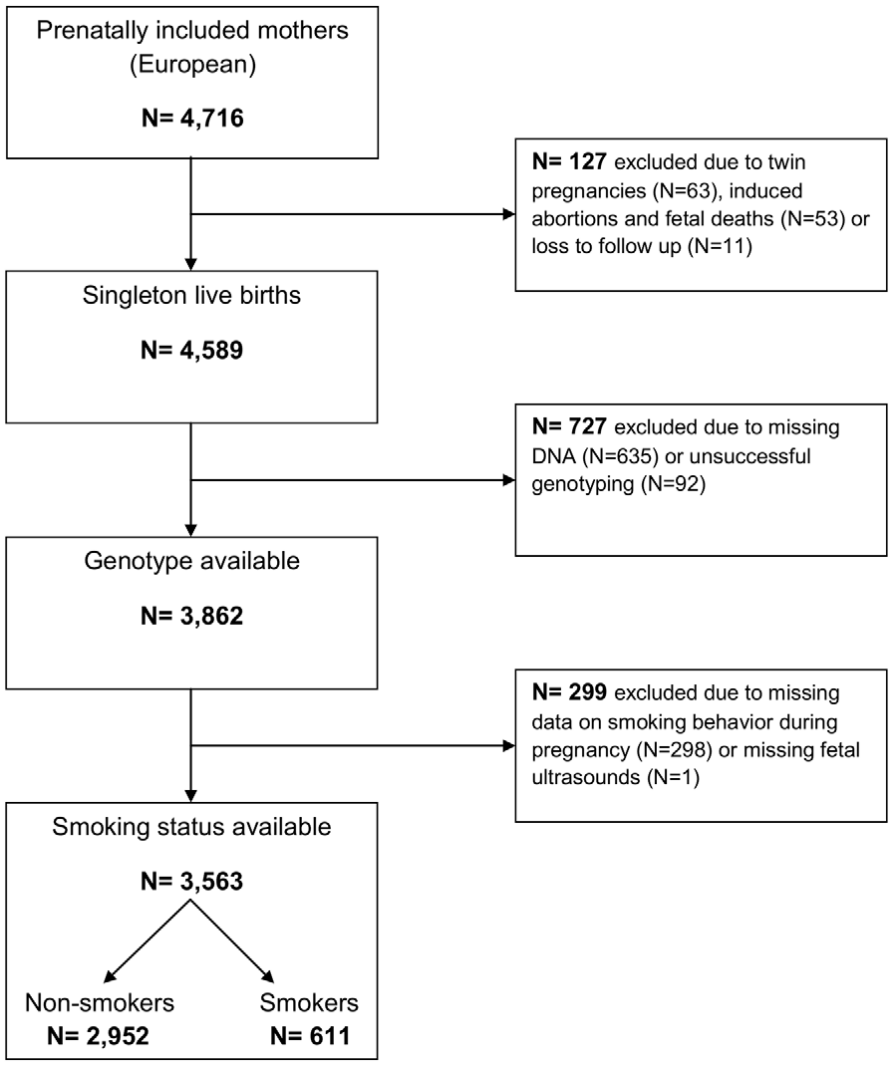
Flow chart of participants included for analysis.

In total, 4,716 mothers of European ancestry (86% Dutch, 14% other European) were included during pregnancy, of whom 3,862 mothers had a singleton live birth and had genotype of rs1051730 available. We had smoking status during pregnancy and fetal ultrasounds available in 3,563 (92.3%) of these mothers. A participant flow chart is given in Figure 2.

### Maternal smoking status

Information about maternal smoking status during pregnancy was obtained by postal questionnaires sent in first, second and third trimester. Response rates for these questionnaires were 91%, 80% and 77%, respectively [18]. Maternal smoking at enrollment was assessed in the first questionnaire by asking the mother whether she smoked during the pregnancy (no, yes, until pregnancy was known). In the second and third trimester questionnaires, the mothers were asked whether they had smoked (no, yes) during second and third trimester, respectively. Mothers who reported to have smoked until pregnancy was known (first trimester only) were considered as non-smokers. Mothers who reported smoking in the second or third questionnaire, were considered as mothers who continued smoking during pregnancy [11]. Among mothers who continued smoking, the number of cigarettes was classified into the following categories: less than 5 cigarettes per day, 5–10 cigarettes per day and more than 10 cigarettes per day.

### Genotyping

Maternal DNA was extracted from whole blood samples. Genotyping of the G>T substitution of rs1051730 in mothers was performed using TaqMan allelic discrimination assay (Applied Biosystems, Foster City, CA, USA). Child DNA was isolated from cord blood. Genotyping was carried out using the Illumina 610 k Quad arrays. The frequency distribution of maternal rs1051730 genotype did not deviate from the Hardy-Weinberg equilibrium in subjects with European ancestry (GG: 45.4%, GT: 43.1%, TT: 11.6%, *P*-value = 0.78). Distribution of fetal genotype was similar (GG: 47.3%, GT: 41.8%, TT: 10.8%, *P*-value = 0.73).

### Fetal growth and birth outcomes

Fetal ultrasound examinations were carried out in two dedicated research centers in each trimester of pregnancy. Reliability and reproducibility of these ultrasound examinations was tested in early pregnancy and was very good (Interobserver intraclass correlation coefficients above 0.988 for all fetal characteristics and interobserver coefficient of variation all between 2.4% and 3.8%) [19]. Fetal ultrasound examinations were used for both establishing gestational age in early pregnancy and assessing second and third trimester fetal growth characteristics. These methods have previously been described in detail [18]. Establishing gestational age by the first day of the last menstrual period is not reliable for a variety of reasons, including the large number of women who do not know their exact date, have irregular menstrual cycles or amenorrhea, use oral contraceptive pills or bleed in early pregnancy [20]–[21]. Pregnancy dating curves were constructed for subjects with complete data on gestational age measured by ultrasonography and the last menstrual period [21]. Crown-rump length was used for pregnancy dating up to until a gestational age of 12 weeks and 5 days (crown-rump length smaller than 65 mm) and biparietal diameter thereafter (gestational age from 12 weeks and 5 days onwards, biparietal diameter larger than 23 mm). Second and third trimester growth measurements (head circumference, abdominal circumference and femur length) were measured to the nearest millimeter using standardized ultrasound procedures [22]. Estimated fetal weight was calculated using the formula by Hadlock [23]. Gestational age adjusted standard deviation scores were constructed for all fetal growth measurements based on reference growth curves from the whole study population [21]. Information on date of birth, sex and birth anthropometrics (head circumference, length and weight) was obtained from community midwife and hospital registries. Gestational age and sex-adjusted standard deviation scores for birth anthropometrics were constructed using growth standards from Usher and McLean [24]. Because head circumference and length were not routinely measured at birth, missing birth measures were completed with data from the first month visit at the routine child health center. Of all measurements, 32% and 19% were based on the first month visit for head circumference and birth length, respectively. No differences in genotype between children with measurements at birth and those with measurements at child health center were observed (Chi-square tests: *P* = 0.67 for head circumference and *P* = 0.51 for birth length).

### Covariates

Maternal age and information about maternal educational level and parity was obtained by a questionnaire at enrollment in the study. Maternal alcohol consumption habits were assessed by questionnaires in each trimester. Maternal anthropometrics, including height and weight, were measured without shoes and heavy clothing and body mass index (BMI) was calculated (weight/height^2^ [kg/m^2^]) at enrollment [25].

### Statistical analysis

Differences in basic characteristics between mothers who did not smoke and who smoked during pregnancy were assessed by using a Student T-test for continuous variables and Chi-square tests for categorical variables. We performed cross-sectional analyses using linear regression models to assess the associations of the maternal risk allele (T-allele of rs1051730) with fetal growth characteristics in second and third trimester (head circumference, femur length, estimated fetal weight) and at birth (head circumference, body length, weight). Based on previous studies, we considered an additive model, but performed a sensitivity analysis using a T-dominant model. All other analyses were based on an additive model and the effect estimates reflect the effect for each additional copy of the risk allele. Analyses were performed in the total group, and in strata of mothers who did not smoke during pregnancy and mothers who smoked during pregnancy. We tested the interaction between maternal smoking and maternal genotype, to assess whether the associations of maternal smoking with fetal growth were modified by the T-allele of rs1051730. All models were adjusted for sex and gestational age at visit. Analyses in the total population were adjusted for smoking status. To assess possible confounding effects, we additionally adjusted for first trimester smoking quantity as a covariate. Next, a fully adjusted model was explored, with further adjustment for maternal age, body mass index at enrollment, parity, educational level and alcohol consumption. The regression models with neonatal head circumference and length as outcome were additionally adjusted for postconceptional age (gestational age at birth for measurements at birth or gestational age at birth plus postnatal age for measurement from the child health centers) and for the source of the measurement (birth or child health center). We repeated the analyses for fetal genotype.

The associations of smoke status, rs1051730 genotype and longitudinally measured fetal growth were analyzed using unbalanced repeated measurement regression models. The repeatedly measured outcome data for this analysis were the gender and gestational age adjusted SD scores. These models take the correlation between repeated measurements of the same participant into account and allow for incomplete outcome data [26]. We have used a fixed effects model without higher order terms. This approach uses the exogenous sampling maximum likelihood to assess the best model to calculate the effect estimates. The genotype categories of non-smokers and smokers categories were included in these models as intercept and as an interaction term with gestational age. Each individual could have a maximum of three measurements per growth characteristic available. The analyses were based on 3,563 subjects with in total 9,751, 9,843 and 10,405 measurements for head circumference, length, and weight, respectively. For all three growth characteristics, the minimum number of measurements was 1, maximum 3 and median was 3 (90% range 2–3) for head circumference, length, and weight. The outcomes reflect the differences in growth in gender and gestational age specific standard deviation scores (SDS). The repeated measurement analysis was performed using the Statistical Analysis System version 9.2 (SAS, Cary, NC), including the Proc Mixed module for unbalanced repeated measurements. All other analyses were performed using the Statistical Package of Social Sciences for Windows (SPSS Inc, Chicago, IL, USA), version 17.0.

## Supporting Information

Table S1 **Subject characteristics of the mothers per maternal genotype (continued smokers n = 610)^1^.** ^1^Values are means (SD) or percentages. ^2^Median (95% range). Differences in distributions between groups were evaluated using a Student T-test for continuous variables and Chi-square tests for categorical variables *P-value<0.05.
(DOC)

Table S2 **Associations of maternal rs1051730 genotype and maternal smoking with longitudinally measured growth measures^1^.** ^1^Values are based on repeated linear regression models and reflect the differences in growth in gender and gestational age specific standard deviation scores (SDS) between the number of risk alleles and smoking status compared to the reference group (SDS = 0). *P-value<0.05; **P-value<0.01.
(DOC)

Table S3 **Cross-sectional associations of maternal rs1051730 genotype with fetal growth characteristics in different trimesters (assuming a T-dominant model)^1^ (n = 3,561).** ^1^Effect estimates (with 95% confidence interval) reflect the differences in phenotype of women with one or two copies of the T-allele of rs1051730, compared to women without risk alleles (assuming a dominant model). ^2^Interaction term = maternal genotype×smoking status. *P-value<0.05; **P-value<0.01. All analyses were adjusted for gestational age at visit, sex and smoking quantity. Analyses in the total group were additionally adjusted for smoking status (yes, no). Birth length and head circumference at birth were additionally adjusted for source of the birth measurements.
(DOC)

Table S4 **Cross-sectional associations of fetal rs1051730 genotype with fetal growth characteristics in different trimesters (adjusted for maternal genotype)^1^ (n = 1,960).** ^1^Effect estimates (with 95% confidence interval) reflect the differences in phenotype for each additional copy of the T-allele of rs1051730 (assuming an additive model). ^2^Interaction term = fetal genotype×smoking status. *P-value<0.05; **P-value<0.01. All analyses were adjusted for maternal rs1051730 genotype, gestational age at visit and sex. Analyses in the total group were additionally adjusted for smoking status (yes, no). Birth length and head circumference at birth were additionally adjusted for source of the birth measurements.
(DOC)

Table S5 **Cross-sectional associations of maternal rs1051730 genotype with fetal growth characteristics in different trimesters^1^ (n = 3,561).** ^1^Effect estimates (with 95% confidence interval) reflect the differences in phenotype for each additional copy of the T-allele of rs1051730 (assuming an additive model). ^2^Interaction term = maternal genotype×smoking status. *P-value<0.05; **P-value<0.01. All analyses were adjusted for gestational age at visit, sex and smoking quantity. Analyses in the total group were additionally adjusted for smoking status (yes, no). Birth length and head circumference at birth were additionally adjusted for source of the birth measurements.
(DOC)

Table S6 **Cross-sectional associations of maternal rs1051730 genotype with fetal growth characteristics in different trimesters^1^ (fully adjusted model n = 3,521).** ^1^Effect estimates (with 95% confidence interval) reflect the differences in phenotype for each additional copy of the T-allele of rs1051730 (assuming an additive model). ^2^Interaction term = maternal genotype×smoking status. *P-value<0.05; **P-value<0.01. All analyses were adjusted for gestational age at visit, sex, maternal age, BMI at enrollment, parity, educational level, alcohol use and smoking quantity. Analyses in the total group were additionally adjusted for smoking status (yes, no). Birth length and head circumference at birth were additionally adjusted for source of the birth measurements.
(DOC)
