# Supplementary material for: A Common Genetic Variant at 15q25 Modifies the Associations of Maternal Smoking during Pregnancy with Fetal Growth: The Generation R Study
Source: PLoS One. 2012 Apr 4;7(4):e34584. doi: 10.1371/journal.pone.0034584 (PMC3319619; doi:10.1371/journal.pone.0034584)
Supplement: Table S1 — Subject characteristics of the mothers per maternal genotype (continued smokers n = 610)1. 1Values are means (SD) or percentages. 2Median (95% range). Differences in distributions between groups were evaluated using a Student T-test for continuous variables and Chi-square tests for categorical variables *P-value<0.05. (DOC) [file pone.0034584.s001.doc]

**Table S1. Subject characteristics of the mothers per maternal genotype (continued smokers n=610)1**

|  | **Total** | **G/G** | **G/T** | **T/T** |
| --- | --- | --- | --- | --- |
|  | **N=610** | **N= 250 (41.0%)** | **N= 289 (47.4%)** | **N= 71 (11.6%)** |
| **Mother** |  |  |  |  |
| Age (years) | 29.7 (5.6) | 29.7 (5.7) | 29.3 (5.6) | 31.5 (4.6) |
| Gestational age at enrolment^2^ (weeks) | 14.1 (9.5 to 29.4) | 14.5 (9.3 to 30.4) | 13.8 (9.5 to 25.5) | 13.5 (9.0 to 28.9) |
| Height (cm) | 169.3 (6.5) | 169.6 (6.6) | 169.6 (6.4) | 167.5 (6.1)* |
| Weight (kg) | 71.2 (14.0) | 71.5 (12.6) | 71.7 (15.1) | 68.4 (13.7) |
| Body mass index (kg/m^2^) | 24.8 (4.5) | 24.9 (4.1) | 24.9 (4.8) | 24.4 (4.7) |
| Parity (% nullipara) | 57.4 | 52.0 | 61.5* | 60.6 |
| Highest education finished (%) |  |  |  |  |
| Primary school | 13.3 | 11.2 | 15.2 | 13.0 |
| Secondary school | 62.5 | 67.5 | 59.2* | 58.0 |
| Higher education | 24.2 | 21.3 | 25.5 | 29.0 |
| Alcohol consumption during pregnancy (% yes) | 61.3 | 59.3 | 60.2 | 72.9* |
| Number of cigarettes smoked (%) |  |  |  |  |
| <5 per day | 45.2 | 45.8 | 45.7 | 41.4 |
| 5-10 per day | 32.7 | 32.5 | 34.3 | 27.1 |
| >10 per day | 22.0 | 21.7 | 20.1 | 31.4 |
| **Child** |  |  |  |  |
| Gestational age at birth^2^ | 40.0 (34.8 to 42.3) | 40.1(33.7 to 42.3) | 39.9 (34.8 to 42.1) | 40.0 (35.9 to 42.7) |
| Birth weight (grams) | 3290 (546) | 3329 (576) | 3286 (526) | 3166 (507)* |
| Sex (% Boys) | 52.5 | 55.6 | 50.5 | 49.3 |
