# Supplementary material for: A Common Genetic Variant at 15q25 Modifies the Associations of Maternal Smoking during Pregnancy with Fetal Growth: The Generation R Study
Source: PLoS One. 2012 Apr 4;7(4):e34584. doi: 10.1371/journal.pone.0034584 (PMC3319619; doi:10.1371/journal.pone.0034584)
Supplement: Table S2 — Associations of maternal rs1051730 genotype and maternal smoking with longitudinally measured growth measures1. 1Values are based on repeated linear regression models and reflect the differences in growth in gender and gestational age specific standard deviation scores (SDS) between the number of risk alleles and smoking status compared to the reference group (SDS = 0). *P-value<0.05; **P-value<0.01. (DOC) [file pone.0034584.s002.doc]

**Table S2. Associations of maternal rs1051730 genotype and maternal smoking with longitudinally measured growth measures**1

|  | **Intercept** | | | | **Growth rate (SDS) / 10 weeks** | | | |
| --- | --- | --- | --- | --- | --- | --- | --- | --- |
|  | **In total population** | | **Among smoking strata** | | **In total population** | | **Among smoking strata** | |
| **Head circumference** | **Intercept** | **95%CI** | **Intercept** | **95%CI** | **Slope** | **95%CI** | **Slope** | **95%CI** |
| Non-smokers, G/G | *Reference* |  | *Reference* |  | *Reference* |  | *Reference* |  |
| Non-smokers, G/T | -0.1639 | -0.3133 to -0.0145* | -0.1639 | -0.3133 to -0.0145* | 0.04646 | 0.0019 to 0.0910* | 0.04646 | 0.0019 to 0.0910* |
| Non-smokers, T/T | -0.2206 | -0.4519 to 0.0106 | -0.2206 | -0.4519 to 0.0106 | 0.08391 | 0.0145 to 0.1534* | 0.08391 | 0.0145 to 0.1534* |
| Smokers, G/G | 0.0536 | -0.2197 to 0.3268 | *Reference* |  | -0.0912 | -0.1744 to -0.0079* | *Reference* |  |
| Smokers, G/T | -0.0998 | -0.3551 to 0.1556 | -0.1505 | -0.4990 to 0.1981 | -0.0624 | -0.1400 to 0.0152 | 0.0285 | -0.0778 to 0.1348 |
| Smokers, T/T | -0.0088 | -0.4888 to 0.4712 | -0.0604 | -0.5994 to 0.4787 | -0.1075 | -0.2534 to 0.0384 | -0.0160 | -0.1799 to 0.1479 |
| **Length growth** |  |  |  |  |  |  |  |  |
| Non-smokers, G/G | *Reference* |  | *Reference* |  | *Reference* |  | *Reference* |  |
| Non-smokers, G/T | -0.0723 | -0.2266 to 0.0821 | -0.0723 | -0.2266 to 0.0821 | 0.0381 | -0.0089 to 0.0852 | 0.0381 | -0.0089 to 0.0852 |
| Non-smokers, T/T | 0.0145 | -0.2222 to 0.2511 | 0.0145 | -0.2222 to 0.2511 | 0.0203 | -0.0520 to 0.0930 | 0.0203 | -0.0520 to 0.0930 |
| Smokers, G/G | 0.2567 | -0.0214 to 0.5348 | *Reference* |  | -0.1390 | -0.2249 to -0.0531** | *Reference* |  |
| Smokers, G/T | 0.2279 | -0.0314 to 0.4871 | -0.02800 | -0.3788 to 0.3229 | -0.1537 | -0.2336 to -0.0739** | -0.0144 | -0.1229 to 0.0942 |
| Smokers, T/T | 0.1053 | -0.3835 to 0.5942 | -0.1502 | -0.6946 to 0.3942 | -0.2138 | -0.3651 to -0.0626** | -0.0743 | -0.2431 to 0.0945 |
| **Weight growth** |  |  |  |  |  |  |  |  |
| Non-smokers, G/G | *Reference* |  | *Reference* |  | *Reference* |  | *Reference* |  |
| Non-smokers, G/T | -0.0918 | -0.2465 to 0.0629 | -0.0918 | -0.2465 to 0.0629 | 0.03770 | -0.0091 to 0.0845 | 0.03770 | -0.0091 to 0.0845 |
| Non-smokers, T/T | -0.0122 | -0.2486 to 0.2241 | -0.0122 | -0.2486 to 0.2241 | 0.03889 | -0.0325 to 0.1103 | 0.03889 | -0.0325 to 0.1103 |
| Smokers, G/G | 0.3417 | 0.0672 to 0.6163* | *Reference* |  | -0.1729 | -0.2559 to -0.0900** | *Reference* |  |
| Smokers, G/T | 0.1231 | -0.1346 to 0.3807 | -0.2172 | -0.5593 to 0.1248 | -0.1209 | -0.1992 to -0.0426** | 0.05276 | -0.0495 to 0.1550 |
| Smokers, T/T | -0.0667 | -0.5495 to 0.4161 | -0.4011 | -0.9310 to 0.1288 | -0.1463 | -0.2935 to -0.0010* | 0.02569 | -0.1334 to 0.1847 |
