# Supplementary material for: A Common Genetic Variant at 15q25 Modifies the Associations of Maternal Smoking during Pregnancy with Fetal Growth: The Generation R Study
Source: PLoS One. 2012 Apr 4;7(4):e34584. doi: 10.1371/journal.pone.0034584 (PMC3319619; doi:10.1371/journal.pone.0034584)
Supplement: Table S4 — Cross-sectional associations of fetal rs1051730 genotype with fetal growth characteristics in different trimesters (adjusted for maternal genotype)1 (n = 1,960). 1Effect estimates (with 95% confidence interval) reflect the differences in phenotype for each additional copy of the T-allele of rs1051730 (assuming an additive model). 2Interaction term = fetal genotype×smoking status. *P-value<0.05; **P-value<0.01. All analyses were adjusted for maternal rs1051730 genotype, gestational age at visit and sex. Analyses in the total group were additionally adjusted for smoking status (yes, no). Birth length and head circumference at birth were additionally adjusted for source of the birth measurements. (DOC) [file pone.0034584.s004.doc]

**Table S4. Cross-sectional associations of fetal rs1051730 genotype with fetal growth characteristics in different trimesters (adjusted for maternal genotype)^1^ (n=1,960)**

|  | **Second trimester** | **Third trimester** | **Birth** |
| --- | --- | --- | --- |
|  | **Head circumference** | **Head circumference** | **Head circumference** |
|  | Difference (95% CI) (mm) | Difference (95% CI) (mm) | Difference (95% CI) (mm) |
| **Total Group** *N=1,959* | 0.33 (-0.14 to 0.79) | 0.20 (-0.47 to 0.87) | 0.57 (-0.63 to 1.78) |
| Non-smokers *N=1,670* | 0.29 (-0.21 to 0.78) | -0.09 (-0.81 to 0.64) | 0.40 (-0.89 to 1.70) |
| Smokers *N=289* | 0.55 (-0.81 to 1.92) | 2.01 (0.17 to 3.84)* | 1.88 (-1.47 to 5.23) |
| **Interaction^2^** | *P=0.91* | *P=0.41* | *P=0.97* |
|  | **Femur length** | **Femur length** | **Body length** |
|  | Difference (95% CI) (mm) | Difference (95% CI) (mm) | Difference (95% CI) (mm) |
| **Total Group** *N=1,959* | 0.02 (-0.12 to 0.16) | -0.02 (-0.19 to 0.15) | 0.70 (-0.97 to 2.37) |
| Non-smokers *N=1,670* | -0.01 (-0.16 to 0.14) | -0.03 (-0.21 to 0.16) | 0.31 (-1.49 to 2.10) |
| Smokers *N=289* | 0.19 (-0.21 to 0.58) | 0.06 (-0.40 to 0.52) | 3.51 (-0.93 to 7.96) |
| **Interaction^2^** | *P=0.90* | *P=0.28* | *P=0.88* |
|  | **Estimated fetal weight** | **Estimated fetal weight** | **Weight** |
|  | Difference (95% CI) (g) | Difference (95% CI) (g) | Difference (95% CI) (g) |
| **Total Group** *N=1,960* | 0.79 (-2.60 to 4.18) | -1.11 (-15.47 to 13.24) | 24.22 (-9.65 to 58.10) |
| Non-smokers *N=1,671* | -0.42 (-4.02 to 3.19) | -4.13 (-19.55 to 11.30) | 17.75 (-18.67 to 54.16) |
| Smokers *N=289* | 8.58 (-1.16 to 18.31) | 19.96 (-18.95 to 58.88) | 67.84 (-24.25 to 159.94) |
| **Interaction^2^** | *P=0.57* | *P=0.49* | *P=0.77* |
