# Supplementary material for: A Common Genetic Variant at 15q25 Modifies the Associations of Maternal Smoking during Pregnancy with Fetal Growth: The Generation R Study
Source: PLoS One. 2012 Apr 4;7(4):e34584. doi: 10.1371/journal.pone.0034584 (PMC3319619; doi:10.1371/journal.pone.0034584)
Supplement: Table S5 — Cross-sectional associations of maternal rs1051730 genotype with fetal growth characteristics in different trimesters1 (n = 3,561). 1Effect estimates (with 95% confidence interval) reflect the differences in phenotype for each additional copy of the T-allele of rs1051730 (assuming an additive model). 2Interaction term = maternal genotype×smoking status. *P-value<0.05; **P-value<0.01. All analyses were adjusted for gestational age at visit, sex and smoking quantity. Analyses in the total group were additionally adjusted for smoking status (yes, no). Birth length and head circumference at birth were additionally adjusted for source of the birth measurements. (DOC) [file pone.0034584.s005.doc]

**Table S5. Cross-sectional associations of maternal rs1051730 genotype with fetal growth characteristics in different trimesters^1^ (n=3,561)**

|  | **Second trimester** | **Third trimester** | **Birth** |
| --- | --- | --- | --- |
|  | **Head circumference** | **Head circumference** | **Head circumference** |
|  | Difference (95% CI)(mm) | Difference (95% CI) (mm) | Difference (95% CI) (mm) |
| **Total Group** *N=3,544* | -0.25 (-0.55 to 0.05) | 0.12 (-0.32 to 0.57) | 0.17 (-0.62 to 0.96) |
| Non-smokers *N=2,940* | -0.23 (-0.55 to 0.10) | 0.23 (-0.25 to 0.71) | 0.31 (-0.55 to 1.17) |
| Smokers *N=604* | -0.38 (-1.16 to 0.40) | -0.37 (-1.48 to 0.75) | -0.61 (-2.66 to 1.45) |
| **Interaction^2^** | *P=0.70* | *P=0.29* | *P=0.43* |
|  | **Femur length** | **Femur length** | **Body length** |
|  | Difference (95% CI)(mm) | Difference (95% CI) (mm) | Difference (95% CI) (mm) |
| **Total Group** *N=3,547* | -0.00 (-0.09 to 0.09) | -0.01 (-0.12 to 0.11) | 0.30 (-0.80 to 1.40) |
| Non-smokers *N=2,943* | 0.04 (-0.05 to 0.14) | 0.08 (-0.04 to 0.20) | 0.82 (-0.38 to 2.02) |
| Smokers *N=604* | -0.24 (-0.46 to -0.01)* | -0.41 (-0.69 to -0.13)** | -2.81 (-5.52 to -0.10)* |
| **Interaction^2^** | *P=0.02* | *P<0.01* | *P=0.03* |
|  | **Estimated fetal weight** | **Estimated fetal weight** | **Weight** |
|  | Difference (95% CI) (g) | Difference (95% CI) (g) | Difference (95% CI) (g) |
| **Total Group** *N=3,561* | 0.31 (-1.85 to 2.46) | 3.07 (-6.22 to 12.34) | 16.28 (-5.27 to 37.82) |
| Non-smokers *N=2,953* | 1.36 (-0.99 to 3.71) | 10.37 (0.22 to 20.51)* | 26.81 (3.05 to 50.57)* |
| Smokers *N=608* | -5.23 (-10.64 to 0.19) | -32.91 (-55.74 to -10.09)* | -37.10 (-88.19 to 13.99) |
| **Interaction^2^** | *P=0.03* | *P<0.01* | *P=0.03* |
